# Individual patient data meta-analysis in cancer.

**DOI:** 10.1038/bjc.1998.339

**Published:** 1998-06

**Authors:** M. Clarke, L. Stewart, J. P. Pignon, L. Bijnens

**Affiliations:** ICRF Clinical Trial Service Unit, Radcliffe Infirmary, Oxford, UK.

## Abstract

As in many areas of health care, treatments for cancer may differ only moderately in their effects on major end points, such as death. But, such differences are worth knowing about, particularly in common diseases in which they could represent a substantial benefit to public health. Large-scale randomized evidence allows moderate differences to be investigated reliably, and one way to achieve this is by meta-analyses of updated and centrally collected individual patient data from all relevant trials. This paper illustrates why this form of research can often be important in cancer. It also offers the first list of such projects, as a source of information on current and past research in this area.


British Journal of Cancer (1998) 77(11), 2036–2044
© 1998 Cancer Research Campaign

### Individual patient data meta-analyses in cancer

**M Clarke[1], L Stewart[2], J-P Pignon[3] and L Bijnens[4]**

[1]ICRF Clinical Trial Service Unit, Radcliffe Infirmary, Oxford OX2 6HE, UK; [2]MRC Cancer Trials Office, 5 Shaftesbury Road, Cambridge, UK; [3]Department of Biostatistics and Epidemiology, Institut Gustave Roussy, 38 rue Camille Desmoulins, 94805 Villejuif Cedex, France; [4]Clinical Data Processing and Systems, Janssen Research Foundation, Turnhoutseweg 30, B-2340 Beerse, Belgium

**Summary** As in many areas of health care, treatments for cancer may differ only moderately in their effects on major end points, such as death. But, such differences are worth knowing about, particularly in common diseases in which they could represent a substantial benefit to public health. Large-scale randomized evidence allows moderate differences to be investigated reliably, and one way to achieve this is by meta-analyses of updated and centrally collected individual patient data from all relevant trials. This paper illustrates why this form of research can often be important in cancer. It also offers the first list of such projects, as a source of information on current and past research in this area.

**Keywords:** individual patient data; meta-analyses; collaborative overview; randomized controlled trial

The conduct of meta-analyses of updated and centrally collected individual patient data (IPD) from all relevant randomized controlled trials has been discussed previously (Stewart and Clarke, 1995). This paper illustrates why this form of research is often particularly important in cancer. It also publishes the first list of IPD meta-analyses in cancer (see Appendix), thereby providing an important source of information on what research has been, or is being, conducted in this area. It may also help avoid duplication of effort as IPD meta-analyses generally involve considerable work, particularly for those who organize them.

### THE NEED FOR SYSTEMATIC REVIEWS TO ASSESS TREATMENTS FOR CANCER

As in many areas of health care, a fundamental principle underlying the need for large-scale randomized evidence in cancer is that, for major end points, the difference between the treatments under investigation is unlikely to be large. But, if a widely practicable treatment produced a moderate improvement for a common disease, this could represent a substantial benefit to public health. Similarly, clear and reliable evidence that there is no such difference could avoid much unnecessary treatment, along with its associated toxicity and cost (Collins et al, 1996).

Large-scale randomized evidence can be obtained by suitably large randomized controlled trials (RCTs) that will accrue future patients, systematic reviews of past trials or ideally a combination of the two. At present, most trials in cancer are of limited size and so this disease is particularly well suited to systematic review. In addition, some treatments have been investigated in numerous RCTs over many years. For example, more than 40 000 women world-wide have, since 1974, been randomized into at least 50 separate trials of tamoxifen vs no tamoxifen for operable breast cancer. Data from approximately 30 000 women in 40 of the trials that began before 1985 were collected and reanalysed by the Early Breast Cancer Trialists' Collaborative Group (EBCTCG) in 1992 to reveal a small, but highly significant, reduction in 10-year mortality for women allocated to tamoxifen (EBCTCG, 1992).

*Received 13 October 1997*
*Revised 18 November 1997*
*Accepted 18 November 1997*

*Correspondence to:* M Clarke

### INDIVIDUAL PATIENT DATA-BASED META-ANALYSES: WHAT ARE THEY?

This and the other meta-analyses conducted by the EBCTCG and other groups listed in the Appendix are based on individual patient data, in which the separate trial results used in the meta-analysis come from central analysis of the raw data from each trial. A limited amount of information on each patient entered into each RCT must be collected centrally, usually by a small secretariat. Any apparent inconsistencies or problems are discussed and, hopefully, resolved by communication with the responsible trialists (Stewart and Clarke, 1995). The finalized data for each trial are then analysed separately to obtain summary statistics that are then combined to give an overall estimate of the effect of treatment. In this way, patients are compared directly only with others in the same trial.

This paper concentrates on IPD meta-analyses in cancer and a list of these has been prepared (see Appendix). IPD meta-analyses have also been performed in other areas of health care, such as antiplatelet therapies in vascular disease (Antiplatelet Trialists' Collaboration, 1994), methotrexate in rheumatoid arthritis (Chernoff et al, 1995) and the effects of exercise in reducing falls and frailty in the elderly (Province et al, 1995). However, reviews based on individual patient data are comparatively rare. Thus, the Cochrane Database of Systematic Reviews (1998), which has existed for some years, is based mainly on published data and only recently has it become possible to incorporate individual patient data. The Appendix does not aim to include any meta-analyses that have been performed with either published data alone or aggregate data supplied by trialists, examples of which go back more than 20 years (Stjernsward, 1974). However, the authors would welcome information on any IPD meta-analyses that have been overlooked and can supply fuller details of those included.

The IPD meta-analyses listed in the Appendix are chiefly concerned with the effects of treatment on relapse or survival rather than factors such as quality of life. Some work has been carried out to combine the results of an IPD meta-analysis of recurrence and survival with average toxicity information from other sources to obtain a clearer idea of the influence of different treatments on a patient's quality of life (Gelber et al, 1996) but, in general, quality of life has not been measured in many trials and, when it has been measured, it is likely to prove difficult to combine the different measures used.

### THE ADVANTAGES OF SYSTEMATIC REVIEWS BASED ON INDIVIDUAL PATIENT DATA TO ASSESS TREATMENTS FOR CANCER

Among the advantages of using IPD in any systematic review are that analyses can include time-to-event calculations; consistently defined patient subgroup and outcome analyses can be performed; and standardized checking and correction procedures can be followed for the data from each trial. It might also be easier for reviewers to obtain additional or updated information on individual patients. These advantages should be true for all forms of health care that can be subjected to systematic review but their relative importance will, of course, vary. Examples of how they have applied in IPD meta-analyses of treatments for cancer are given below.

### Time to event analyses

This major advantage of collecting IPD can be obtained even if an absolute minimum amount of data is collected, namely the allocated treatment and the time interval to the outcome under investigation. Typically, more data than these are sought on each randomized patient, but even this minimum would allow summary statistics based on the entire survival experience to be calculated and a survival curve to be constructed. In cancer trials and reviews, the primary outcome of interest is often death and so these analyses may reveal an important prolongation of survival, which might not be apparent if follow-up data for a fixed point in time were collected.

For example, the IPD that were collected for a meta-analysis of platinum-based combination chemotherapy vs single non-platinum drugs in the treatment of advanced ovarian cancer showed that a reliance on 2-year survival data would exaggerate the difference between the two treatments. As Table 1 shows, the improvements in survival for patients allocated to combination chemotherapy compared with those allocated to a single drug are clearly at their greatest at 2 years and subsequently there is little difference between the two treatments (Stewart and Parmar, 1993). Aggregate data could, of course, be requested from each trial for each time point to perform these analyses, but this approach will give less sensitive results, especially when the event rate is high.

### Consistency of effect in patient subgroups

In small trials and reviews, subgroup or multiple outcome analyses may lead to misleading conclusions but if large-scale randomized evidence is available then this can be used, with appropriate caution, in determining whether the differences between treatments are greater for particular groups of patients. However, any such analyses should, ideally, be regarded as hypothesis-generating, for testing in future studies. If subgroup analyses are to be performed, they need to be as complete as possible and should preferably involve commonly defined subgroups and outcomes across all of the trials in a review. This will rarely be possible if the review is based solely on the published literature as, regardless of the problems associated with not being able to include unpublished trials, the information that has been published on various subgroup analyses may well be incomplete and is probably biased. Although trialists could be asked to fill in a table containing aggregate data on different types of patient and of outcome, this might prove difficult for many trialists, particularly for those with no data management or statistical support. In addition, if the outcome data had also to be supplied for different lengths of follow-up, the necessary tables could potentially contain more cells than patients in a trial. To complete such a table, the trialists would also need to adopt the centrally determined definitions for subgroups and outcomes. Thus, the collection of IPD may prove simpler for the trialists. It also allows the secretariat to prepare the necessary files for analysis and to apply consistent subgroup and outcome definitions across the included trials.

For example, in acute leukaemia, it is usual to distinguish between children and adults both in trials and in clinical practice generally. However, the definition of a child varies between trialists. Some trials in the USA have included patients up to 21 years of age (van Eys et al, 1989), whereas childhood trials in the UK are often restricted to those aged 14 years and younger (Chessells et al, 1995). A consequent advantage of an IPD meta-analysis is that a common definition of such a patient characteristic can be used across all the trials. This has recently been performed to investigate the duration and intensity of therapy for childhood acute lymphoblastic leukaemia (Childhood ALL Collaborative Group, 1996). It is also possible that the data collected for an IPD meta-analysis could be used to investigate the effects of treatment if varying definitions are used for a particular subgroup.

Leukaemia also provides a useful example of when there may be a variety of definitions for a particular outcome. This is so with 'event-free survival', in which the outcomes considered as 'events' may vary between trial groups. If the relevant data on each possible contributing event are collected from each trial then a common definition can be adopted (Childhood ALL Collaborative Group, 1996). Again, the IPD meta-analysis might also allow for the presentation of results from each of the contributing events separately so that the reader of the review can obtain an estimate of the relative effects of treatment on whatever they define as 'event-free survival'.

### Checking and correction of data

Although our combined experience is that fraud in trials appears to be rare; errors, misunderstandings or inadvertently inappropriate analyses are not and the ability to check the individual patient data may reveal problems or misunderstandings that can be resolved through consultation with the trialists. This can be particularly important in the treatment of cancer as such trials tend to run over relatively long periods of time and are, therefore, particularly susceptible to design changes during their course. As an example, it might be decided that one of the treatments in a two-arm trial should be stopped and so allocations to that arm are closed even though accrual may continue to the other treatment group. Only the patients who were concurrently randomized in such a study should be analysed for comparative purposes, but this is not always done. If the individual patient data are available then this can be rectified (Birch et al, 1988; Pignon et al, 1992). In addition,

© Cancer Research Campaign 1998

**Table 1** Survival differences at fixed time points for 1283 patients in 11 trials of platinum-based combination chemotherapy vs single non-platinum drugs in advanced ovarian cancer (Stewart and Parmar, 1993)

| Follow-up (years) | Odds ratio | Absolute improvement in survival (%) | *P*-value |
|---|---|---|---|
| 1 | 0.87 | 3.2 | 0.23 |
| 2 | 0.76 | 6.2 | 0.02 |
| 3 | 0.88 | 2.3 | 0.31 |
| 4 | 0.92 | 1.2 | 0.56 |
| 5 | 0.94 | 0.8 | 0.69 |

checking of the raw data might reveal that a trial had used a quasi-random method of allocation (e.g. alternation or odd/even birth date) and, because of the danger that such schemes can lead to bias, these trials might then be excluded from the meta-analysis.

### Obtaining unpublished additional data

As already noted, death is often one of the most important measures of outcome in cancer trials. In malignancies with very poor prognosis, a large proportion of patients will die relatively quickly and follow-up of a few years may be sufficient to obtain a reliable estimate of the relative efficacy of the treatments under investigation. However, in some diseases (such as early-stage breast or prostate cancer), many patients who will eventually die of their disease might live several years beyond diagnosis and primary treatment. It could then be many years or decades before a reliable estimate of the mortality effects of the different treatments can be made. This may be true for overall mortality, but it might be especially true for cause-specific mortality (or for the incidence of second cancers) if the treatments have long-term hazards.

IPD meta-analyses are a means by which this can be investigated and may be the best way for some long-term effects to be assessed. By periodically collecting updated data on each patient, the amount of follow-up can be continually extended and if the end point of interest is death this can sometimes be achieved through the use of national mortality records, either by the trialists or by the reviewer (Stewart and Clarke, 1995). Similarly, cancer registries might be used to help collect data on the diagnosis of second cancers. If a central database has already been prepared that contains the necessary baseline variables for each patient, it is a relatively easy task to add this type of additional follow-up information, if it is available, before conducting new analyses.

As an example of the importance of continuing to extend the follow-up in a disease such as breast cancer, the collaborators in the EBCTCG overview were, in 1990, sent a questionnaire asking them to predict the 10-year survival rates, after having seen the 5-year results in 1985 (EBCTCG, 1988). Of the 78 trialists who responded, none predicted that the treatments given during the first few years after diagnosis would produce additional benefits between years 5 and 10 as great as those actually observed (Clarke and Stewart, 1994).

As well as providing unexpected findings on overall mortality during longer follow-up, an IPD meta-analysis might also allow the investigation of cause-specific mortality (e.g. long-term fatal side-effects). This can be particularly important in cancer when long-term hazards are quite possible because of the biological mechanisms of the treatments used. Although observational studies may help to investigate these, systematic reviews of the relevant randomized trials from far enough in the past will have the substantial benefit of the removal of systematic bias. One such investigation has been performed (Cuzick et al, 1994) for a subset of the trials of radiotherapy after surgery for breast cancer, in which detailed information on causes of death was sought. This found an excess of cardiovascular deaths in the period more than 10 years after primary treatment for women allocated to receive radiotherapy. These trials used old radiotherapy techniques so it is unclear how directly applicable the findings are to modern techniques. But, it was still important to find that radiotherapy can produce such hazards and the individual trials were too small to investigate this reliably.

### DISCUSSION

The two ways of collecting large-scale randomized evidence: large prospective randomized trials and systematic reviews of past trials are complementary. Systematic reviews that contain IPD meta-analyses might generate hypotheses about particular interventions or subgroups for testing in future trials and they can also foster international collaboration that might help to facilitate the conduct of randomized trials that are sufficiently large to investigate reliably any promising new treatments. For example, two large trials were started (Le Pechoux et al, 1995; Stephens et al, 1996) after the IPD meta-analysis of chemotherapy in non-small-cell lung cancer (Non-Small Cell Lung Cancer Collaborative Group, 1995).

### CONCLUSION

This report highlights the need for reviewers to consider whether systematic reviews in cancer should involve updated and centrally collected data on each and every randomized patient. An IPD meta-analysis may require more time and resources than some other techniques for systematic review, but it should lead to a more reliable assessment of the treatments under investigation.

### ACKNOWLEDGEMENTS

We are grateful to the researchers involved in IPD meta-analyses in cancer who provided information on their projects. We would also like to thank Marc Buyse, Liz Greaves, Pascal Piedbois, Lennarth Nyström and Richard Peto for their comments on earlier versions of this manuscript. MC is an ICRF research scientist at the Clinical Trial Service Unit, Oxford, UK; LS is a senior scientist at the MRC Cancer Trials Office, Cambridge, UK, and an Honorary Visiting Fellow at the UK Cochrane Centre; JPP is senior biostatistician at the Institut Gustave-Roussy, France; and LB is senior statistician at Janssen Research Foundation but worked on this paper while at the EORTC Data Center, Brussels, Belgium.

### REFERENCES

Advanced Bladder Overview Collaboration (1995) Does neoadjuvant chemotherapy improve the survival of patients with locally advanced bladder cancer? A meta-analysis of individual patient data from randomised clinical trials. *Br J Urol* **75**: 206–213

Advanced Colorectal Cancer Meta-analysis Project (1992) Modulation of 5-fluorouracil by leucovorin in patients with advanced colorectal cancer: evidence in terms of response rate. *J Clin Oncol* **10**: 896–903

Advanced Colorectal Cancer Meta-analysis Project (1994) Meta-analysis of randomised trials testing the biochemical modulation of 5-fluorouracil by methotrexate in metastatic colorectal cancer. *J Clin Oncol* **12**: 960–969

Advanced Ovarian Cancer Trialists Group (1991) Chemotherapy in advanced ovarian cancer: an overview of randomised clinical trials. *Br Med J* **303**: 884–893

Anon (1984) Review of mortality results in randomised trials in early breast cancer. *Lancet* **2**: 1205

© Cancer Research Campaign 1998

Antiplatelet Trialists' Collaboration (1994) Collaborative overview of randomised trials of antiplatelet therapy – I: prevention of death, myocardial infarction, and stroke by prolonged antiplatelet therapy in various categories of patients. *Br Med J* **308**: 81–106

Arriagada R, Pignon JP, Laplanche A and Le Chevalier T (1997) Prophylactic cranial irradiation for small-cell lung cancer. *Lancet* **349**: 138

Birch R, Omura GA, Greco FA and Perez CA (1988) Patterns of failure in combined chemotherapy and radiotherapy for limited small cell lung cancer: Southeastern Cancer Study Group experience. *NCI Monograph* **6**: 265–270

Chernoff MC, Wang M, Anderson JJ and Felson DT (1995) Problems and suggested solutions in creating an archive of clinical trials data to permit later meta-analysis: an example of methotrexate trials in rheumatoid arthritis. *Controlled Clin Trials* **16**: 342–355

Chessells JM, Bailey C, Richards SM, for the Medical Research Council Working Party on Childhood Leukaemia (1995) Intensification of treatment and survival in all children with lymphoblastic leukaemia: results of UK Medical Research Council trial UKALL X. *Lancet* **345**: 143–148

Childhood ALL Collaborative Group (1996) Duration and intensity of maintenance chemotherapy in acute lymphoblastic leukaemia: overview of 42 trials involving 12 000 randomised children. *Lancet* **347**: 1783–1788

Chronic Myeloid Leukemia Trialists' Collaborative Group (1997) Interferon alfa versus chemotherapy for chronic myeloid leukemia: a meta-analysis of seven randomised trials. *J Natl Cancer Inst* **89**: 1616–1620

Clahsen P, van de Velde C, Goldhirsch A, Rossbach J, Sertoli M, Bijnens L, Sylvester R and co-operating investigators (1995) An overview of randomized perioperative polychemotherapy trials in early breast cancer. *Proc Annu Meet Am Soc Clin Oncol* **14**: 129 (a214)

Clahsen PC, van de Velde CJH, Goldhirsch A, Rossbach J, Sertoli M, Bijnens L, Sylvester R and co-operative investigators (1998) An overview of randomized perioperative polychemotherapy trials in women with early breast cancer. *J Clin Oncol* (in press)

Clarke MJ and Stewart LA (1994) Obtaining data from randomised controlled trials: how much do we need to perform reliable and informative meta analyses? *Br Med J* **309**: 1007–1010

Clarke M, Gray R, Dunn J and MacLennan I (1992) Combination chemotherapy for myelomatosis. *Lancet* **340**: 433

Cochrane Database of Systematic Reviews (1998) In *The Cochrane Library* (database on disk and CDROM), The Cochrane Collaboration. Update Software: Oxford

Collins R, Peto R, Gray R and Parish S (1996) Large-scale randomized evidence: trials and overviews. In *Oxford Textbook of Medicine*, Weatherall DJ, Ledingham JGG and Warrell DA. (eds), pp. 21–32. Oxford University Press: Oxford

Cuzick J, Stewart H, Peto R, Fisher B, Kaae S, Johansen H, Lythgoe JP and Prescott RJ (1987*a*) Overview of randomized trials comparing radical mastectomy without radiotherapy against simple mastectomy with radiotherapy in breast cancer. *Cancer Treat Rep* **71**: 7–14

Cuzick J, Stewart H, Peto R, Baum M, Fisher B, Host H, Lythgoe JP, Ribeiro G, Scheurlen H and Wallgren A (1987*b*) Overview of randomized trials of postoperative adjuvant radiotherapy in breast cancer. *Cancer Treat Rep* **71**: 15–29

Cuzick J, Stewart H, Rutqvist L, Houghton J, Edwards R, Redmond C, Peto R, Baum M, Fisher B, Host H, Lythgoe J, Ribeiro G and Scheurlen H (1994) Cause-specific mortality in long-term survivors of breast cancer who participated in trials of radiotherapy. *J Clin Oncol* **12**: 447–453

Early Breast Cancer Trialists' Collaborative Group (1988) Effects of adjuvant tamoxifen and of cytotoxic therapy on mortality in early breast cancer: an overview of 61 randomised trials among 28 896 women. *N Engl J Med* **319**: 1681–1692

Early Breast Cancer Trialists' Collaborative Group (1990) *Treatment of Early Breast Cancer*, Vol 1. *Worldwide Evidence 1985–1990*. Oxford University Press: Oxford

Early Breast Cancer Trialists' Collaborative Group (1992) Systemic treatment of early breast cancer by hormonal, cytotoxic, or immune therapy: 133 randomised trials involving 31 000 recurrences and 24 000 deaths among 75 000 women. *Lancet* **339**: 1–15, 71–85

Early Breast Cancer Trialists' Collaborative Group (1995) Effects of radiotherapy and surgery in early breast cancer: an overview of the randomised trials. *N Engl J Med* **333**: 1444–1455

Early Breast Cancer Trialists' Collaborative Group (1996) Ovarian ablation in early breast cancer: an overview of the randomised trials. *Lancet* **348**: 1189–1196

Gelber RD, Cole BF, Goldhirsch A, Rose C, Fisher B, Osborne CK, Boccardo F, Gray R, Gordon NH, Bengtsson NO and Sevelda P (1996) Adjuvant chemotherapy plus tamoxifen compared with tamoxifen alone for postmenopausal breast cancer: meta-analysis of quality-adjusted survival. *Lancet* **347**: 1066–1071

International Hodgkin's Disease Collaborative Group (1998) The influence of more extensive radiotherapy and adjuvant chemotherapy on long-term outcome of early stage Hodgkin's disease: a meta-analysis of 23 randomized trials involving 3888 patients. *J Clin Oncol* **16**: 830–843

Larsson LG, Andersson I, Bjurstom N, Fagerberg G, Frisell J, Tabár L and Nyström L (1997) Updated overview of the Swedish randomized trials on breast cancer screening with mammography: age group 40–49 at randomization. *Monogr Natl Cancer Inst* **22**: 57–61

Le Pechoux C, Cojean I, Arriagada R, Pignon JP, Auquier A, Tarayre M and Le Chevalier T (1995) From the results of the meta-analysis evaluating the role of chemotherapy in non-small cell lung cancer (NSCLC) to the IALT project. *Eur J Cancer* **30a** (suppl. 5); S221

Liver Infusion Meta-analysis Group (1997) Portal vein chemotherapy for colorectal cancer: a meta-analysis of 4000 patients in 10 studies. *J Natl Cancer Inst* **89**: 497–505

Loeffler M, Brosteanu O, Hasenclaver D, Sextro M, Assouline D, Bartolucci AA, Cassileth PA, Crowther D, Diehl V, Fisher RI, Hoppe RT, Jacobs P, Pater JL, Pavlovsky S, Thompson E and Wiernik P for the IDHD Overview study group (1998) Meta-analysis of chemotherapy versus combined modality treatment trials in Hodgkin's disease. *J Clin Oncol* **16**: 818–829

Meta-analysis Group in Cancer (1996) Reappraisal of hepatic artery infusion in the treatment of non-resectable liver metastases from colorectal cancer. *J Natl Cancer Inst* **88**: 252–258

Non-Small Cell Lung Cancer Collaborative Group (1995) Chemotherapy in non-small cell lung cancer: a meta-analysis using updated individual patient data from 52 randomised clinical trials. *Br Med J* **311**: 899–909

Nyström L, Rutqvist LE, Wall S, Lindgren A, Lindqvist M, Rydén S, Andersson I, Bjurstam N, Fagerberg G, Frisell J, Tabár L and Larsson LG (1993) Breast cancer screening with mammography: overview of Swedish randomised trials. *Lancet* **341**: 973–978

Ovarian Cancer Meta-analysis Project (1991) Cyclophosphamide plus cisplatin versus cyclophosphamide, doxorubicin and cisplatin chemotherapy of ovarian carcinoma: a meta-analysis. *J Clin Oncol* **9**: 1668–1674

Pawinski A, Sylvester R, Bouffioux C, Kurth KH, Parmar MKB and Bijnens L (1996) A combined analysis of EORTC/MRC randomized clinical trials for the prophylactic treatment of TaT1 bladder cancer. *J Urol* **156**: 1934–1941

Pignon JP, Arriagada R, Ihde DC, Johnson DH, Perry MC, Souhami RL, Brodin O, Joss RA, Kies MS, Lebeau B, Onoshi T, Østerlind K, Tattersall MHN and Wagner H (1992) A meta-analysis of thoracic radiotherapy for small-cell lung cancer. *N Engl J Med* **327**: 1618–1624

Pignon JP, Bourhis J and Domenge C, on behalf of the MACH-NC secretariat (1995) Meta-analysis of chemotherapy in head and neck cancer: individual patient data versus literature data. *Br J Cancer* **72**: 1062

Prostate Cancer Trialists' Collaborative Group (1995) Maximum androgen blockade in advanced prostate cancer: an overview of 22 randomised trials with 3283 deaths in 5710 patients. *Lancet* **346**: 265–269

Province MA, Hadley EC, Hornbrook MC, Lipstiz LA, Miller JP, Mulrow CD, Ory MG, Sattin RW, Tinetti ME and Wolf SL, for the FICSIT Group (1995) The effects of exercise on falls in elderly patients: a preplanned meta-analysis of the FICSIT trials. *JAMA* **273**: 1341–1347

Sarcoma Meta-analysis Collaboration (1997) Adjuvant chemotherapy for localized resectable soft-tissue sarcoma of adults: meta-analysis of individual data. *Lancet* **350**: 1647–1654

Sinclair D, Greaves L, Clarke M and Baigent C (1995) International register of current protocols in childhood acute lymphoblastic leukaemia. *Eur J Cancer* **31** (suppl.5): s114

Specht L and Gray R, for the HD Collaborative Group (1991) Overview of randomized trials of adjuvant combination chemotherapy and of extended field radiotherapy in nodal Hodgkin's disease. *Proc Eur Conf Clin Oncol Cancer Nurs* **6**: s236 (a1445)

Specht L and Gray R (1996) Meta-analysis of randomized trials of more extensive radiotherapy and of adjuvant combination chemotherapy in early stage Hodgkin's disease. *Proc Annu Meet Am Soc Clin Oncol* **15**: 410 (a1250)

Stephens RJ, Parmar MKB, Souhami RL and Spiro S (1996) Chemotherapy in non-small cell lung cancer. Large trial will reduce uncertainty. *Br Med J* **312**: 248

Stewart LA and Parmar MKB (1993) Meta-analysis of the literature or of individual patient data: is there a difference? *Lancet* **341**: 418–422

Stewart L and Clarke M, for the Cochrane Collaboration Working Group on meta-analyses using individual patient data (1995) Practical methodology of meta-analyses (overviews) using updated individual patient data. *Stat Med* **14**: 2057–2079

© Cancer Research Campaign 1998

Stjernsward J (1974) Decreased survival related to irradiation postoperatively in early operable breast cancer. *Lancet* **2**: 1285–1286

Tierney JF, on behalf of the Sarcoma Meta-analysis Collaboration (1996) A meta-analysis using individual patient data from randomised clinical trials of adjuvant chemotherapy for soft tissue sarcoma. *J Clin Oncol* **14**: 1751 (a2024)

van Eys J, Berry D, Crist W, Doering E, Fernbach D, Pullen J, Shuster J, and Wharam M (1989) A comparison of two regimens for high-risk acute lymphocytic leukemia in childhood. *Cancer* **63**: 23–29

Wheatley K, on behalf of the AML Collaborative Group (1995) Meta-analysis of randomized trials of idarubicin or mitozantrone versus daunorubicin as induction therapy for acute myeloid leukaemia. *Blood* **86**(suppl. 1): 434a (a1724)

### APPENDIX

### IPD meta-analyses of treatments or screening for cancer that have assessed remission, relapse or survival unless otherwise stated (listed alphabetically by cancer site)

| Cancer | Review group | Scope of the project | Brief details of the project | Contact address |
|---|---|---|---|---|
| Bladder | Advanced Bladder Cancer Overview Collaboration (ABCOC) | Randomized trials of local therapy vs neoadjuvant or concurrent chemotherapy in locally advanced bladder cancer | IPD were sought from all trials of neoadjuvant or concurrent chemotherapy in locally advanced bladder cancer. The main end point was survival, and subgroup analyses by age, sex, stage and grade were performed. Results were published in 1995 (Advanced Bladder Overview Collaboration, 1995). The meta-analysis will be updated during 1998/99. | ABCOC, MRC CTO, 5 Shaftesbury Road, Cambridge CB2 2BW, UK |
| Bladder | EORTC and MRC combined analysis | Randomized trials of prophylactic treatment of TaT1 bladder cancer | IPD are being collected and preliminary results of a combined analysis of the EORTC and MRC trials has been published (Pawinski et al, 1996). Plans are being made to expand the meta-analysis to include all relevant trials. | EORTC, Meta-analysis Unit, EORTC Data Center, Avenue E Mounier 83, Boite 11, 1200 Bruxelles, Belgium |
| Breast | Cuzick et al | Randomized trials of radiotherapy vs none which began before 1975 and of radical mastectomy vs simple mastectomy with radiotherapy | IPD were collected in the early 1980s and the results on long-term survival, were published first in 1987 (Cuzick et al, 1987*a* and *b*). Further follow-up and more detailed information, including whether the tumour was in the left or the right breast and the cause of death for women dying more than 10 years after primary treatment, were collected and published subsequently for the unconfounded trials of radiotherapy (Cuzick et al, 1994). | Dr Jack Cuzick, ICRF, PO Box 123, Lincoln's Inn Field, London WC2A 3PX, UK |
| Breast | Early Breast Cancer Trialists' Collaborative Group (EBCTCG) | Randomized trials of any aspect of the treatment of early (i.e. operable) breast cancer, which had survival as the primary end point | IPD were first sought for trials of tamoxifen or chemotherapy vs control in 1984 (Anon, 1984). The results of these treatments on 5-year survival were published in brief (EBCTCG, 1988), and as a monograph containing additional information (EBCTCG, 1990). Further follow-up, and data from all other trials that had assessed treatments for early breast cancer were collected in 1989/90 and the new analyses have been published (EBCTCG, 1992 and 1995). A third cycle was initiated, and the preliminary analyses were presented to the trialists, in 1995. The results for the ovarian ablation trials have been published (EBCTCG, 1996), and other papers have been submitted. The overview takes place every 5 years and the fourth cycle will begin in 1999 for a meeting of trialists in 2000. | EBCTCG, CTSU, Radcliffe Infirmary, Oxford OX2 6HE, UK |
| Breast | Perioperative chemotherapy trialists | Randomized trials of perioperative chemotherapy in early breast cancer | IPD have been collected on both perioperative monochemotherapy and polychemotherapy. An abstract reporting the results of a meta-analysis of the latter was published in 1995 (Clahsen et al, 1995) and a manuscript has been accepted for publication (Clahsen et al, 1998). | EORTC, Meta-analysis Unit (address as above) |
| Breast | Nyström et al | Randomized trials of mammographic screening | Seven randomized trials of mammographic screening have been identified, but only four of these evaluated mammography alone and all these were performed in Sweden. In 1987, an IPD meta-analysis was initiated to compare women who were invited for screening with those who were not invited, using breast cancer mortality as the end point. Results from the first follow-up were published in 1993 (Nyström et al, 1993). Further follow-up was collected and an updated report has been published (Larsson et al, 1997). | Dr Lennarth Nyström, Department of Epidemiology and Public Health, Umeå University, S-901-85 Umeå, Sweden |

 *© Cancer Research Campaign 1998*

| Cancer | Review group | Scope of the project | Brief details of the project | Contact address |
|---|---|---|---|---|
| Breast | EORTC | Randomized trials of LHRH-agonist and tamoxifen treatment of metastatic breast cancer | The protocol for this project is currently being finalized. | EORTC, Meta-analysis Unit (address as above) |
| Colorectal | Liver Infusion Meta-analysis Group | Randomized trials of continuous, post-operative portal vein infusion of chemotherapy lasting some days vs control | IPD were collected for trials beginning before 1987 and the results were published in 1997 (Liver Infusion Meta-analysis Group, 1997). | Meta-Analysis Group in Cancer (MAGIC), Department of Oncology, Hôpital Henri Mondor, 51 av du Maréchal de Lattre de Tassigny, 94 000 Créteil, France, and Colorectal Cancer Collaboration, CTSU (address as above) |
| Colorectal | Colorectal Cancer Collaboration | Randomized trials of any aspect of the primary or adjuvant treatment of any type of colon or rectal cancer in which there might be some hope of cure | IPD were collected for trials beginning before 1987 and the preliminary analyses were presented to the trialists in 1993. Additional data have since been collected, before the preparation of the appropriate manuscripts. | Colorectal Cancer Collaboration, CTSU (address as above) |
| Colorectal, advanced | MAGIC, FUFOL | Randomized trials of 5-FU alone vs 5-FU + folinic acid | A protocol was sent to all investigators in October 1990. IPD were collected from December 1990 to April 1991 and preliminary analyses were presented to trialists in May 1991. A manuscript was circulated among trialists later that year and was published in 1992 (Advanced Colorectal Cancer Meta-analysis Project, 1992). | Meta-Analysis Group in Cancer (MAGIC) (address as above) |
| Colorectal, advanced | MAGIC, FUMTX | Randomized trials of 5-FU alone vs 5-FU + methotrexate | A protocol was sent to all investigators in October 1991. IPD were collected from December 1991 to April 1993 and preliminary analyses were presented to trialists in May 1993. A manuscript was circulated among trialists later that year and was published in 1994 (Advanced Colorectal Cancer Meta-analysis Project, 1994). | Meta-Analysis Group in Cancer (MAGIC) (address as above) |
| Colorectal, advanced | MAGIC, MAIA | Randomized trials of intravenous 5-FU or FUDR vs hepatic artery infusion 5-FU | A protocol was sent to all investigators in October 1993. IPD were collected from December 1993 to December 1994 and preliminary analyses were presented to trialists in May 1995. A manuscript was circulated among trialists later that year and was published in 1996 (Meta-analysis Group in Cancer, 1996). A pharmacoeconomic study is ongoing. | Meta-Analysis Group in Cancer (MAGIC) (address as above) |
| Colorectal, advanced | MAGIC/IGR, FUCONT | Randomized trials of 5-FU bolus vs 5-FU continuous infusion | A protocol was sent to all investigators in August 1994. IPD were collected from November 1994 to April 1996 and preliminary analyses were presented to trialists in May 1996. A manuscript is in preparation. | Meta-Analysis Group in Cancer (MAGIC) (address as above) and Dr Jean-Pierre Pignon, Department of Biostatistics, Institut Gustave-Roussy – IGR, rue Camille Desmoulins, 94805 Villejuif Cedex, France |
| Glioma | Ongoing | Randomized trials of chemotherapy | A protocol for this project was circulated to trialists in June 1997. | Meta-analysis Group MRC CTO (address as above) |
| Head and neck | Meta-analysis of Chemotherapy in Head and Neck Cancer Collaborative Group (MACH-NC-CG) | Randomized trials of chemo-therapy vs none in squamous cell carcinoma receiving locoregional treatment, of chemotherapy and organ preservation(Pignon et al, 1995), and ofdifferent timings of the same radiochemotherapy combinations | IPD were collected for trials that started after 1965 and were completed before 1994. The main end point is survival. Subgroup analyses by age, sex, performance status, site of primary tumour and stage are planned. Preliminary results werepresented to trialists in January 1997 and a manuscript will be submitted in 1997. A second cycle of the meta-analysis is planned. | Dr Jean-Pierre Pignon (address as above) |

© Cancer Research Campaign 1998

| Cancer | Review group | Scope of the project | Brief details of the project | Contact address |
|---|---|---|---|---|
| Head and neck | Currently in design | Randomized trials comparing conventional radiotherapy to modified radiotherapy fractionation in squamous cell carcinoma | This meta-analysis is currently at the planning stage, with discussion taking place between the IGR Biostatistics Department and the EORTC Meta-analysis Unit. It will be coordinated on behalf of an international collaborative group, and IPD will be sought from all relevant trials. | Dr Jean-Pierre Pignon (address as above) and EORTC Meta-analysis Unit (address as above) |
| Hodgkin's disease | International Hodgkin's Disease Collaborative Group | Randomized trials assessing either chemotherapy after radiotherapy, or the extent of radiotherapy for early-stage Hodgkin's disease | Aggregate data were collected in 1990 and a draft report was circulated to trialists (Specht et al, 1991). It was agreed that this should not be submitted until the analyses had been improved by the use of IPD. These were collected (Specht and Gray, 1996) and the results have been published (InternationalHodgkin's Disease Collaborative Group, 1998). | Dr Lena Specht, Department of Oncology, Herlev Hospital, DK-2730 Herlev, Denmark |
| Hodgkin's disease | International Database on Hodgkin's Disease Overview Study Group | Randomized trials of combined modality treatment vs chemotherapy alone for intermediate- or advanced-stage Hodgkin's disease | IPD have been collected since 1993 and the results have been published (Loeffler et al, 1998). | Dr Markus Loeffler, IMISE, University of Leipzig, D-04103 Leipzig, Germany |
| Leukaemia, acute lympho-blastic (ALL) | Childhood ALL Collaborative Group | Randomized trials of various aspects of the treatment of childhood ALL | IPD were collected from trials assessing different durations of maintenance therapy, and preliminary analysis was presented to the trialists in 1991. IPD were subsequently collected for other aspects of maintenance or intensification therapy, and the results of these analyses have been published (Childhood ALL Collaborative Group, 1996). This overview has also led to the production of a uniquely comprehensive annual register of ongoing randomized trials in the treatment of this disease (Sinclair et al, 1995). The next cycle of the overview will concentrate on CNS-directed therapies and anthracyclines. Data collection will begin in 1998. | Childhood ALL Collaborative Group CTSU (address as above) |
| Leukaemia, acute myeloid (AML) | AML Collaborative Group (AMLCG) | Randomized trials of various aspects of the treatment of AML | IPD were collected for trials comparing different anthracyclines or assessing different doses of cytosine arabinoside in 1994. Preliminary results have been presented for the former (Wheatley et al, 1995) and a full manuscript has been submitted. Further data were collected for the latter, along with IPD for trials assessing growth factor support, the intensification of therapy, or the duration of maintenance and were presented to the AMLCG in 1996. The next cycle of the overview will concentrate mainly on growth factors and preliminary analyses will be presented in 1998. | AMLCG CTSU (address as above) |
| Leukaemia, acute myeloid (AML) | AML Collaborative Group (AMLCG) | Randomized trials of autologous bone marrow transplantation (A-BMT) vs control or chemotherapy | IPD have been collected and preliminary analyses were presented to the AMLCG in 1995 and 1996. | EORTC Meta-analysis Unit (address as above) and AMLCG CTSU (address as above) |
| Leukaemia, chronic lymphocytic (CLL) | CLL Trialists' Collaborative Group | Randomized trials of various aspects of the treatment of CLL | IPD were collected from trials comparing early vs deferred treatment, the addition of prednisone, or CHOP therapy vs other chemotherapy and preliminary analyses were presented to the trialists in 1993. Further follow-up information has been collected and a manuscript will be submitted in 1998. | CLL Trialists' Collaborative Group CTSU (address as above) |
| Leukaemia, chronic myeloid (CML) | CML Trialists' Collaborative Group | Randomized trials of various aspects of the treatment of CML | IPD were collected from trials assessing the use of interferon, or comparing hydroxyurea vs busulphan, and preliminary analyses were presented in 1995; the results have been published (Chronic Myeloid Leukemia Trialists' Collaborative Group, 1997). | CML Trialists' Collaborative Group CTSU (address as above) |
| Lung, non-small-cell | Non-Small Cell Lung Cancer Collaborative Group | Randomized trials of chemotherapy | IPD were sought from all trials that started after 1965 and were completed before 1992. The main end point was survival and subgroup analyses by age, sex, stage, histology and performance status | NSCLCG MRC CTO (address as above) and |

© Cancer Research Campaign 1998

| Cancer | Review group | Scope of the project | Brief details of the project | Contact address |
|---|---|---|---|---|
| | | | were performed. Results were presented to trialists in 1993 and published in 1995 (Non-Small Cell Lung Cancer Collaborative Group, 1995). The meta-analysis will be updated during 1998/99. | Dr Jean-Pierre Pignon (address as above) |
| Lung, non-small-cell | PORT Collaborative Group | Randomized trials of post-operative radiotherapy | This project was initiated in 1996. IPD have been requested from all trials that started after 1965 and were completed before 1996. The main end points are survival and progression-free survival. Subgroup analyses by age, sex, stage, histology and performance status were carried out. Results were presented to trialists in August 1997. A manuscript will be published in 1998. | PORT MRC CTO (address as above) |
| Lung, small-cell | Small Cell Lung Cancer Meta-Analysis Group | Randomized trials of thoracic radiotherapy vs none in limited stage small-cell lung cancer treated by chemotherapy | IPD were collected for trials beginning before 1989. The main end point was survival and subgroup analyses by age, sex and performance status were performed. Results were presented to trialists in 1991 and published in 1992 (Pignon et al, 1992). New data including further follow-up are being collected. | Dr Jean-Pierre Pignon (address as above) |
| Lung, small-cell | Prophylactic Cranial Irradiation Overview Collaborative Group (PCIO-CG) | Randomized trials of prophylactic cranial irradiation vs none, in complete responders (Arriagada et al, 1997) | IPD are being collected for trials beginning before 1996. The main end point is survival. The secondary end points are disease-free survival, time to brain metastases, time to other metastases and time to locoregional recurrence. Subgroup analyses by age, sex, performance status, treatment use to obtain complete response and extension of initial disease are planned. Preliminary results were presented to trialists in August 1997. | Dr Jean-Pierre Pignon (address as above) |
| Melanoma | MAGIC/EORTC | Randomized trials of any adjuvant treatment of any type of malignant melanoma | A protocol was sent to all investigators in May 1995. IPD are being collected. | Meta-Analysis Group in Cancer (MAGIC) (address as above) and EORTC Meta-analysis Unit (address as above) |
| Multiple myeloma | Myeloma Trialists' Collaborative Group | Randomized trials of any aspect of the treatment of multiple myeloma | IPD were collected in 1993 (Clarke et al, 1992) and preliminary analyses have been presented to the trialists. A manuscript has been submitted for the comparison of combination chemotherapy vs melphalan and prednisone. Further IPD are being collected for trials of interferon vs control and preliminary results were presented to the relevant trialists in 1997. In addition, the IPD (from nearly 13 000 patients) might be used to develop a new prognostic staging system. | Myeloma Trialists' Collaborative Group CTSU (address as above) |
| Non-Hodgkin's lymphoma | Ongoing | Randomized trials of interferon | A protocol has been prepared and data are being collected. | Dr Ama Rohatiner and Dr Walter Gregory, Medical Oncology, St Bartholomew's Hospital, 45 Little Britain, West Smithfield, London EC1A 7BE, UK |
| Non-Hodgkin's lymphoma | EORTC | Randomized trials of regimens containing either doxorubicin or mitoxantrone | A summary of the protocol has been sent to all collaborators and the protocol itself is now being prepared. | EORTC Meta-analysis Unit (address as above) and Dr Magnus Björkholm and Dr Eva Ösby, Department of Medicine, Karolinska Hospital, Stockholm, Sweden |
| Oesophageal | Oesophageal Cancer Collaborative Group (OCCG) | Randomized trials of preoperative radiotherapy in oesophageal cancer | IPD were sought from all trials that were completed by 1994. The main end-point was survival and subgroup analyses were performed by age, sex and tumour location. The results were presented to trialists in 1995 and updated in 1996. A paper has been submitted for publication. | OCCG MRC CTO (address as above) |

| Cancer | Review group | Scope of the project | Brief details of the project | Contact address |
|---|---|---|---|---|
| Ovarian | Advanced Ovarian Cancer Trialists' Group (AOCTG) | Randomized trials of chemotherapy in advanced ovarian cancer comparing single-agent vs combination non-platinum therapies, single non-platinum vs platinum combinations, non-platinum combinations vs the same combination plus platinum, single vs combination platinum, or cisplatin vs carboplatin | IPD were sought from all relevant trials of chemotherapy in advanced ovarian cancer. Results were presented to trialists in 1990 and published in 1991 (Advanced Ovarian Cancer Trialists Group, 1991). Further data were collected in 1995, and updated analyses for all but the first comparison were performed. These included subgroup analyses of age, stage, performance status, extent of operation, residual tumour bulk, histological cell type and grade for the cisplatin vs carboplatin trials. A manuscript has been submitted for publication. An associated project investigating dose intensity in cisplatin vs carboplatin trials is ongoing. | AOCTG MRC CTO (address as above) |
| Ovarian | Ovarian cancer meta-analysis analysis project | Randomized trials comparing cyclophosphamide plus cisplatin vs the same plus doxorubicin | Results were published in 1991 (Ovarian Cancer Meta-analysis Project, 1991). | Meta-Analysis Group in Cancer (MAGIC) (address as above) |
| Ovarian | Currently in design | Randomized trials of paclitaxel | This meta-analysis is currently in design but IPD will be sought for all relevant trials. The primary end point will be survival though a limited amount of quality-of-life data may also be collected. | Meta-analysis Group MRC CTO (address as above) |
| Prostate | Prostate Cancer Trialists' Collaborative Group (PCTCG) | Randomized trials of any aspect of the treatment of prostate cancer | IPD were collected from trials assessing maximum androgen blockade in advanced prostate cancer and the results have been published (Prostate Cancer Trialists' Collaborative Group, 1995). The project has been extended to all randomized trials and preliminary analyses were presented to the trialists in 1997. | PCTCG, Biometrics Department, Netherlands Cancer Institute, Plesmanlaan 121, 1066 CX Amsterdam, The Netherlands |
| Soft tissue sarcoma | Sarcoma Meta-analysis Collaboration | Randomized trials of adjuvant chemotherapy | IPD were sought from all trials that started after 1970. The principal end point was survival with local recurrence-free interval, distant recurrence-free interval, recurrence-free interval and recurrence-free survival as additional end points. Subgroup analyses were performed by age, sex, disease site, histology, grade, tumour size, primary therapy and extent of resection. Results were presented to collaborators in 1995 and at ASCO in 1996 (Tierney et al, 1996). These were updated in 1996 and a manuscript has been submitted for publication. | Meta-analysis Group MRC CTO (address as above) |
| Solid and non-solid tumours | MAGIC | Randomized trials of any chemotherapy vs the same plus G-CSF | A protocol was sent to all investigators in December 1994. IPD are being collected. | Meta-Analysis Group in Cancer (MAGIC) (address as above) |
| Uterine cervix | Currently in design | Randomized trials of neoadjuvant chemotherapy | This meta-analysis is currently at the planning stage, with discussions taking place between the MRC Meta-analysis Group and the EORTC Meta-analysis Unit. It will be coordinated on behalf of an international collaborative group, and IPD will be sought from all relevant trials. | Meta-analysis Group MRC CTO (address as above) and EORTC Meta-analysis Unit (address as above) |

© Cancer Research Campaign 1998


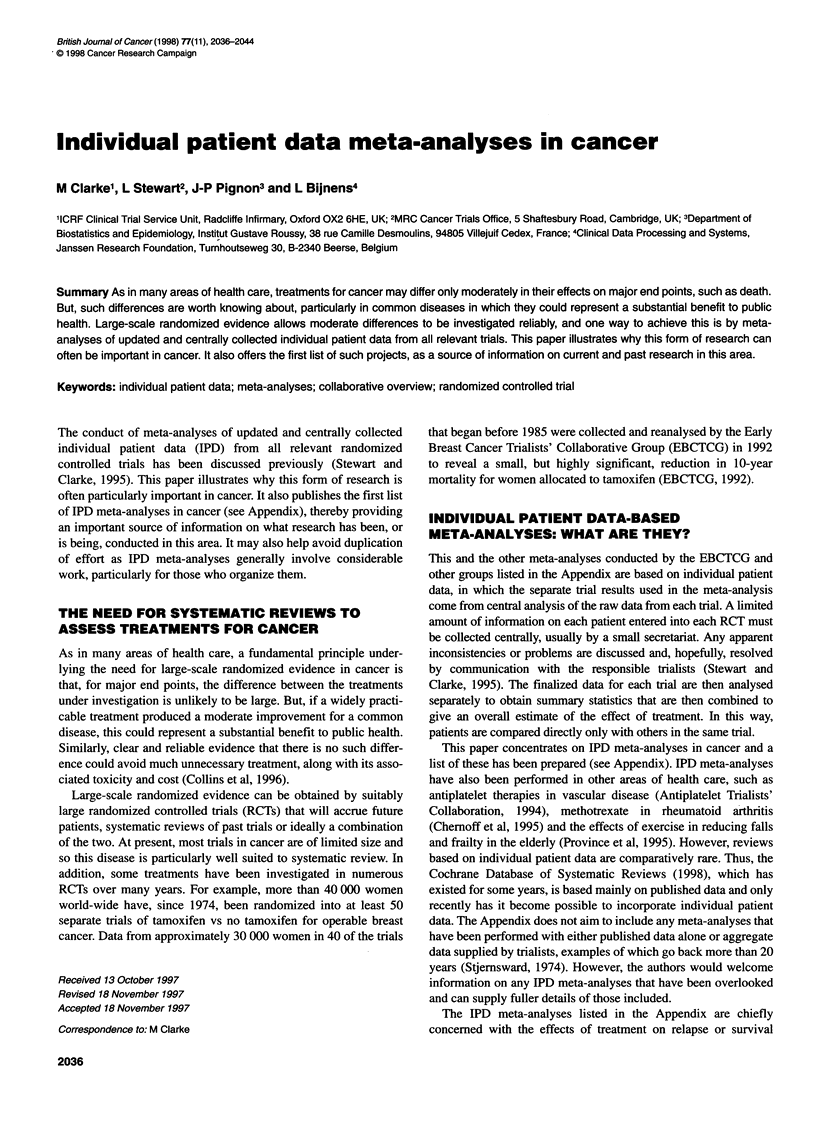

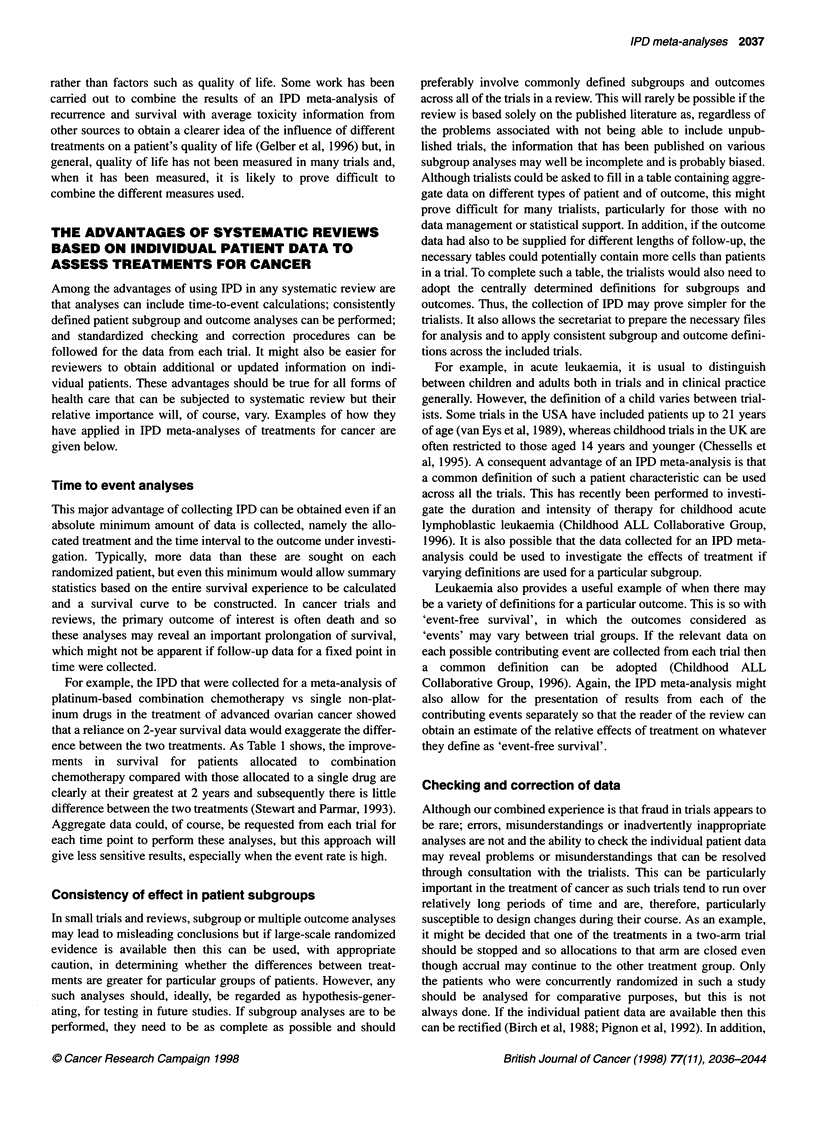

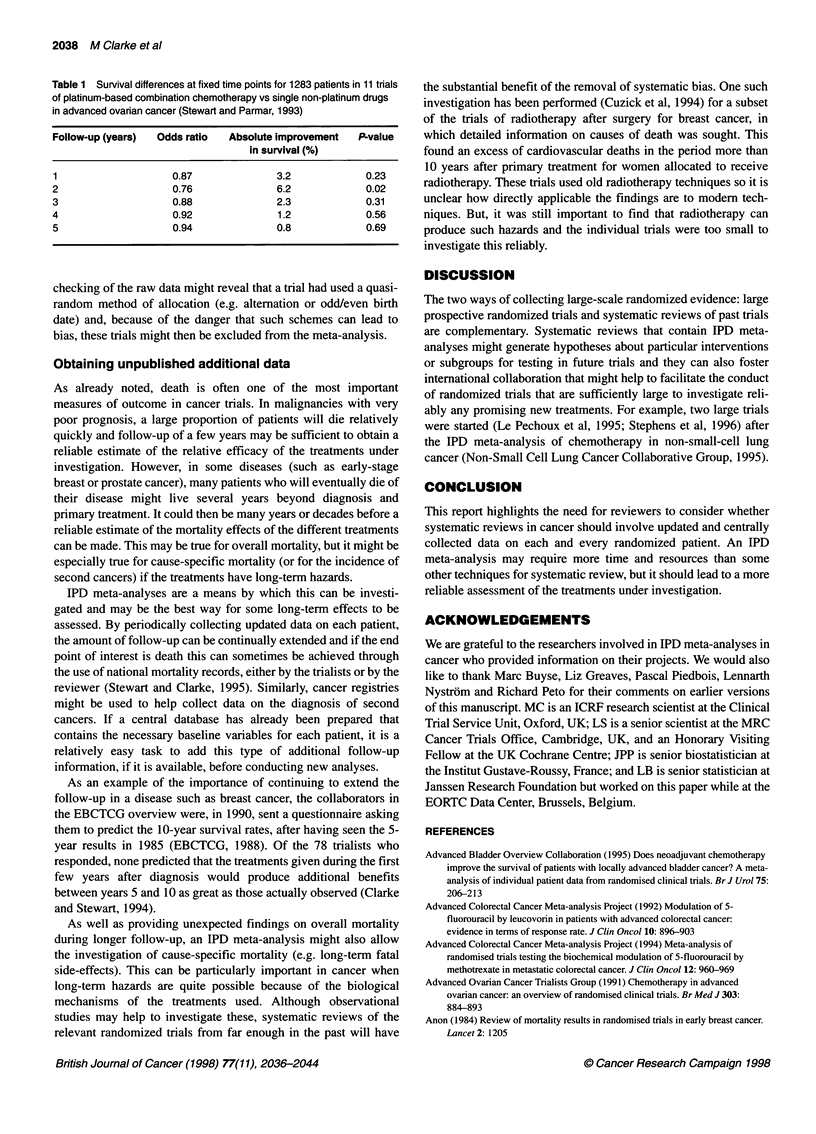

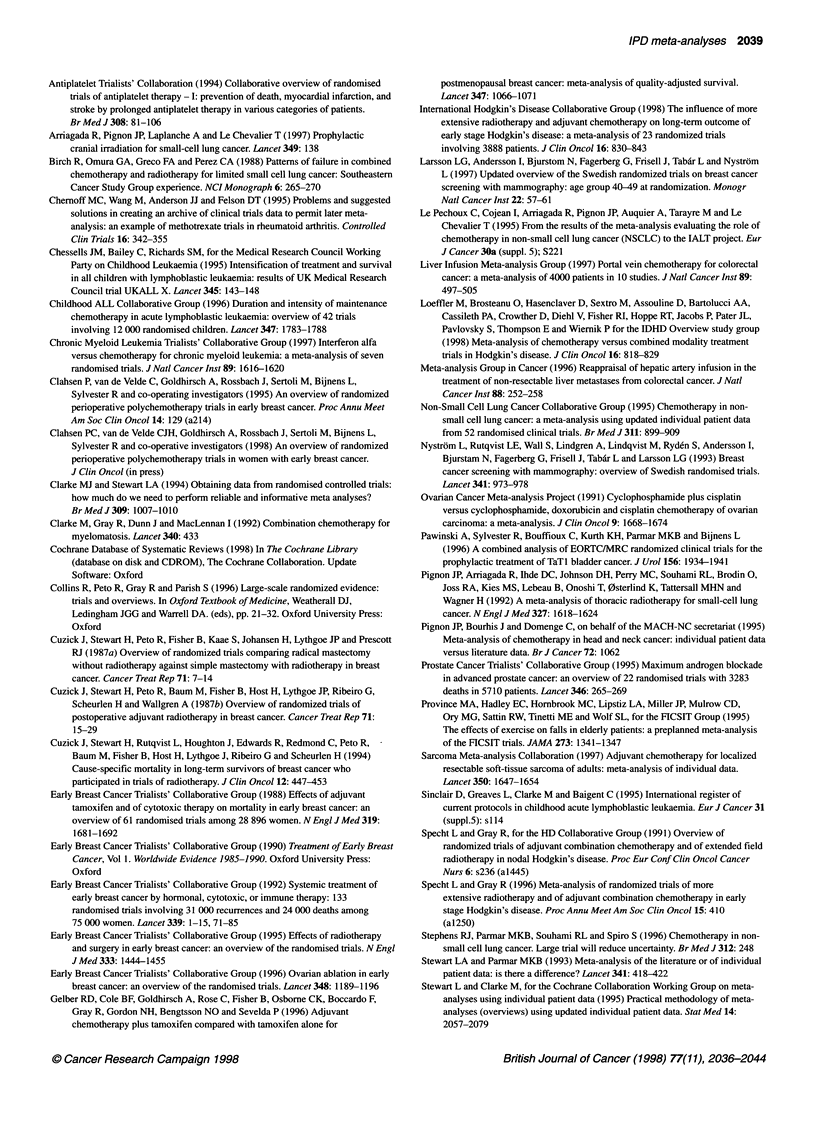

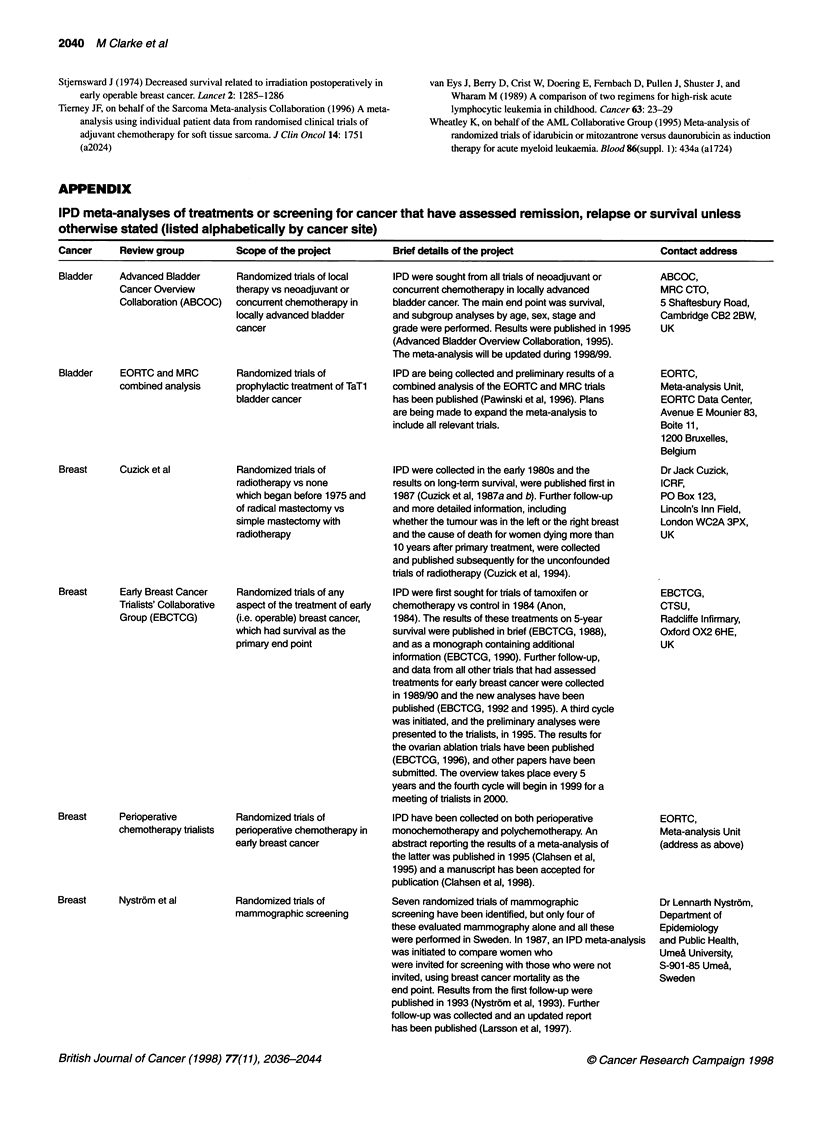

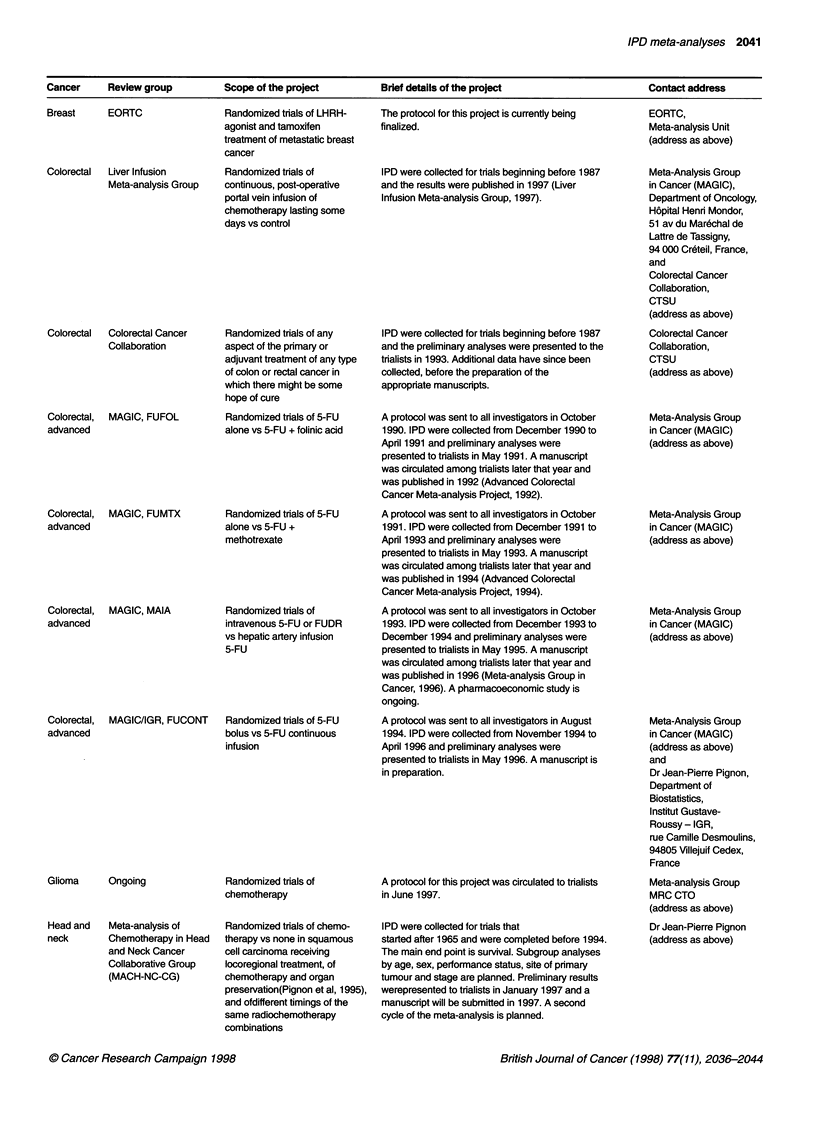

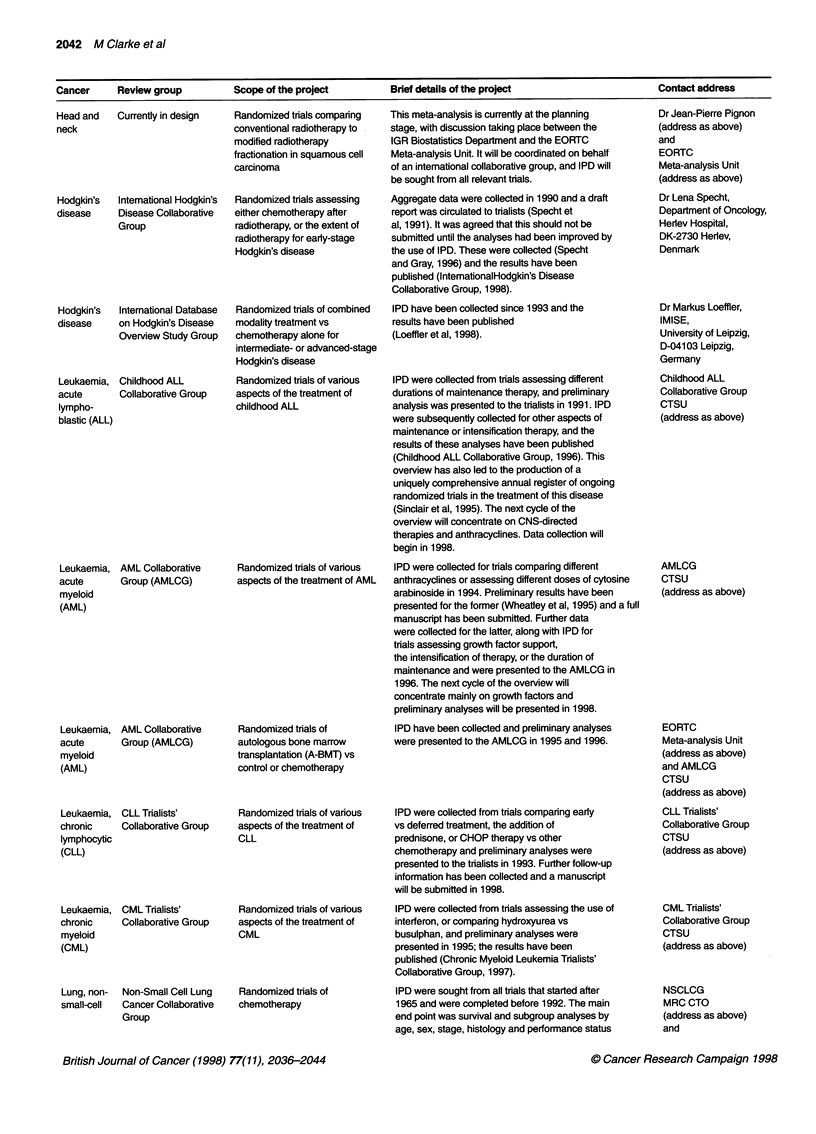

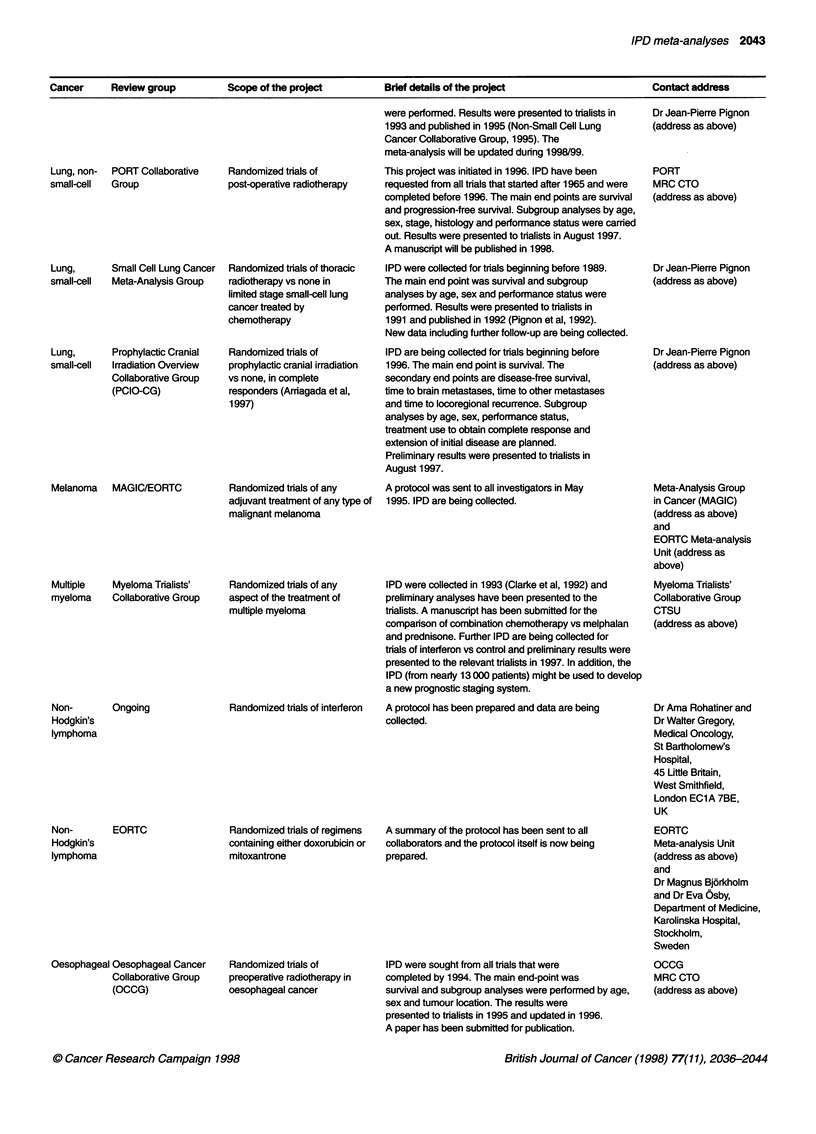

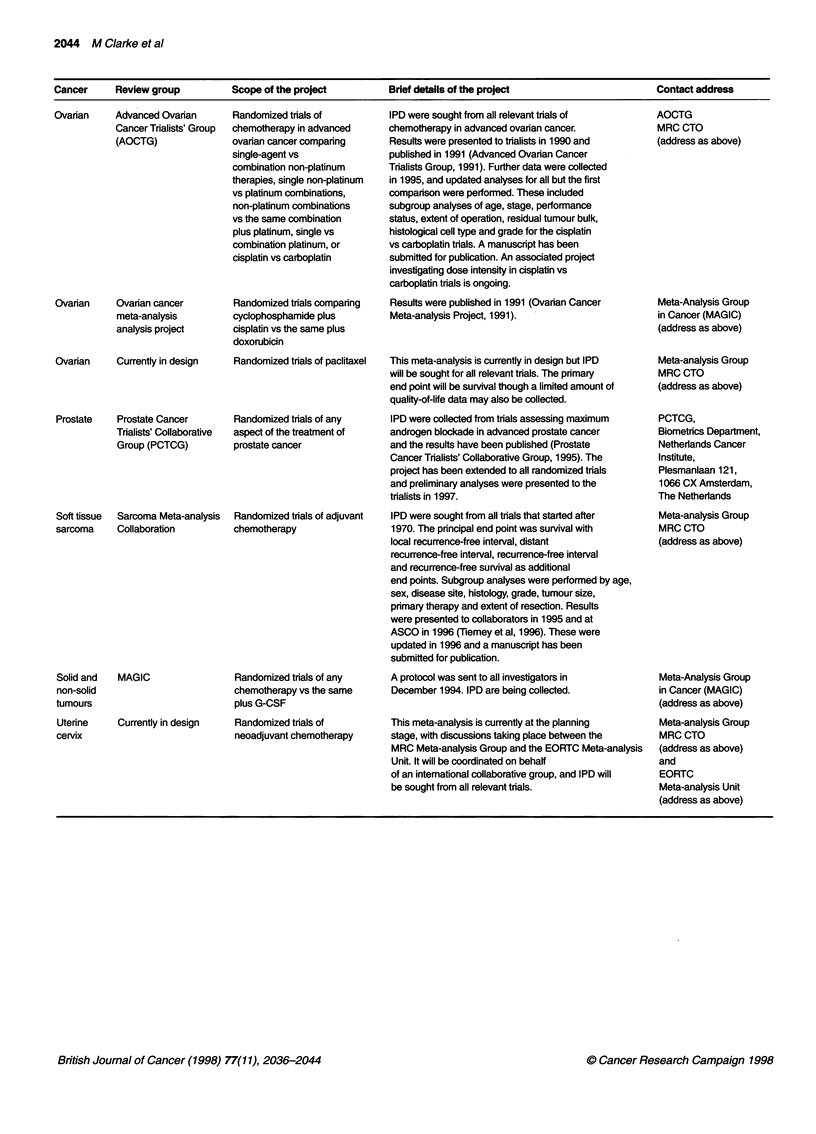


## References

[OCR_00387] Arriagada R., Pignon J. P., Laplanche A., Le Chevalier T. (1997). Prophylactic cranial irradiation for small-cell lung cancer.. Lancet.

[OCR_00391] Birch R., Omura G. A., Greco F. A., Perez C. A. (1988). Patterns of failure in combined chemotherapy and radiotherapy for limited small cell lung cancer: Southeastern Cancer Study Group experience.. NCI Monogr.

[OCR_00396] Chernoff M. C., Wang M., Anderson J. J., Felson D. T. (1995). Problems and suggested solutions in creating an archive of clinical trials data to permit later meta-analysis: an example of methotrexate trials in rheumatoid arthritis.. Control Clin Trials.

[OCR_00403] Chessells J. M., Bailey C., Richards S. M. (1995). Intensification of treatment and survival in all children with lymphoblastic leukaemia: results of UK Medical Research Council trial UKALL X. Medical Research Council Working Party on Childhood Leukaemia.. Lancet.

[OCR_00431] Clarke M. J., Stewart L. A. (1994). Obtaining data from randomised controlled trials: how much do we need for reliable and informative meta-analyses?. BMJ.

[OCR_00436] Clarke M., Gray R., Dunn J., MacLennan I. (1992). Combination chemotherapy for myelomatosis.. Lancet.

[OCR_00459] Cuzick J., Stewart H., Peto R., Baum M., Fisher B., Host H., Lythgoe J. P., Ribeiro G., Scheurlen H., Wallgren A. (1987). Overview of randomized trials of postoperative adjuvant radiotherapy in breast cancer.. Cancer Treat Rep.

[OCR_00450] Cuzick J., Stewart H., Peto R., Fisher B., Kaae S., Johansen H., Lythgoe J. P., Prescott R. J. (1987). Overview of randomized trials comparing radical mastectomy without radiotherapy against simple mastectomy with radiotherapy in breast cancer.. Cancer Treat Rep.

[OCR_00466] Cuzick J., Stewart H., Rutqvist L., Houghton J., Edwards R., Redmond C., Peto R., Baum M., Fisher B., Host H. (1994). Cause-specific mortality in long-term survivors of breast cancer who participated in trials of radiotherapy.. J Clin Oncol.

[OCR_00499] Gelber R. D., Cole B. F., Goldhirsch A., Rose C., Fisher B., Osborne C. K., Boccardo F., Gray R., Gordon N. H., Bengtsson N. O. (1996). Adjuvant chemotherapy plus tamoxifen compared with tamoxifen alone for postmenopausal breast cancer: meta-analysis of quality-adjusted survival.. Lancet.

[OCR_00513] Larsson L. G., Andersson I., Bjurstam N., Fagerberg G., Frisell J., Tabár L., Nyström L. (1997). Updated overview of the Swedish Randomized Trials on Breast Cancer Screening with Mammography: age group 40-49 at randomization.. J Natl Cancer Inst Monogr.

[OCR_00532] Loeffler M., Brosteanu O., Hasenclever D., Sextro M., Assouline D., Bartolucci A. A., Cassileth P. A., Crowther D., Diehl V., Fisher R. I. (1998). Meta-analysis of chemotherapy versus combined modality treatment trials in Hodgkin's disease. International Database on Hodgkin's Disease Overview Study Group.. J Clin Oncol.

[OCR_00550] Nyström L., Rutqvist L. E., Wall S., Lindgren A., Lindqvist M., Rydén S., Andersson I., Bjurstam N., Fagerberg G., Frisell J. (1993). Breast cancer screening with mammography: overview of Swedish randomised trials.. Lancet.

[OCR_00560] Pawinski A., Sylvester R., Kurth K. H., Bouffioux C., van der Meijden A., Parmar M. K., Bijnens L. (1996). A combined analysis of European Organization for Research and Treatment of Cancer, and Medical Research Council randomized clinical trials for the prophylactic treatment of stage TaT1 bladder cancer. European Organization for Research and Treatment of Cancer Genitourinary Tract Cancer Cooperative Group and the Medical Research Council Working Party on Superficial Bladder Cancer.. J Urol.

[OCR_00565] Pignon J. P., Arriagada R., Ihde D. C., Johnson D. H., Perry M. C., Souhami R. L., Brodin O., Joss R. A., Kies M. S., Lebeau B. (1992). A meta-analysis of thoracic radiotherapy for small-cell lung cancer.. N Engl J Med.

[OCR_00574] Pignon J. P., Bourhis J. (1995). Meta-analysis of chemotherapy in head and neck cancer: individual patient data vs literature data.. Br J Cancer.

[OCR_00582] Province M. A., Hadley E. C., Hornbrook M. C., Lipsitz L. A., Miller J. P., Mulrow C. D., Ory M. G., Sattin R. W., Tinetti M. E., Wolf S. L. (1995). The effects of exercise on falls in elderly patients. A preplanned meta-analysis of the FICSIT Trials. Frailty and Injuries: Cooperative Studies of Intervention Techniques.. JAMA.

[OCR_00611] Stephens R. J., Parmar M. K., Souhami R. L., Spiro S. (1996). Chemotherapy in non-small cell lung cancer. Large trial will reduce uncertainty. Steering Committee of the Big Lung Trial.. BMJ.

[OCR_00619] Stewart L. A., Clarke M. J. (1995). Practical methodology of meta-analyses (overviews) using updated individual patient data. Cochrane Working Group.. Stat Med.

[OCR_00615] Stewart L. A., Parmar M. K. (1993). Meta-analysis of the literature or of individual patient data: is there a difference?. Lancet.

[OCR_00629] Stjernswärd J. (1974). Decreased survival related to irradiation postoperatively in early operable breast cancer.. Lancet.

[OCR_00639] van Eys J., Berry D., Crist W., Doering E., Fernbach D., Pullen J., Shuster J., Wharam M. (1989). A comparison of two regimens for high-risk acute lymphocytic leukemia in childhood. A Pediatric Oncology Group Study.. Cancer.

